# Is an Incidental Meckel's Diverticulum Truly Benign?

**DOI:** 10.1155/2015/679097

**Published:** 2015-02-09

**Authors:** Caroline C. Jadlowiec, Jennifer Bayron, William T. Marshall

**Affiliations:** ^1^University of Connecticut Integrated General Surgery Residency Program, Farmington, CT 06030, USA; ^2^Saint Francis Hospital and Medical Center, Hartford, CT 06105, USA

## Abstract

Meckel's diverticulum is the most common congenital abnormality of the gastrointestinal tract and it is found to affect nearly 2 percent of the population. Interestingly, the surgical management of an asymptomatic Meckel's diverticulum remains widely controversial in the adult population. Review of the literature finds the overall risk of Meckel's diverticulum becoming symptomatic to be low; however, the risk accompanying its resection also proves to be minimal thus perpetuating the question of its proper management. We report our experience with an elderly patient who required an emergent operative intervention and was incidentally found to have Meckel's diverticulum. Review of final pathology found Meckel's diverticulum to contain a carcinoid tumor. In our review, the presence of a carcinoid tumor within Meckel's diverticulum is a rare finding, but its incidence may further support the resection of incidentally found asymptomatic Meckel's diverticulum in patients of all ages.

## 1. Introduction

Meckel's diverticulum is the most common congenital abnormality of the gastrointestinal tract. Affecting nearly 2 percent of the population, the surgical management of an incidentally found Meckel's diverticulum in the adult population remains controversial. This controversy is minimal in the pediatric population where resection is encouraged secondary to the seemingly significant risk of developing complications in the first two years of life. We report our experience with an elderly patient who developed a transverse colon volvulus which resulted in operative intervention with discovery and resection of an incidentally found Meckel's diverticulum. On final pathology, the resected Meckel's was found to contain a carcinoid tumor. The finding of a carcinoid tumor within a Meckel's diverticulum is a rarely reported event and, consequently, makes standardization of surgical management challenging. Review of the adult literature finds the risk of lifetime complications arising from a Meckel's to range anywhere from 2–6 percent with similar percentages shown to accompany the surgical morbidity of resection (2–12%) [1, 2]. Accordingly, in reviewing our own experience and likewise acknowledging improved surgical outcomes within the last decade, it appears worthwhile to discuss what is known regarding the incidental Meckel's diverticulum and perhaps reconsider surgical resection in patients of all ages.

## 2. Case Presentation

**Figure 1:**
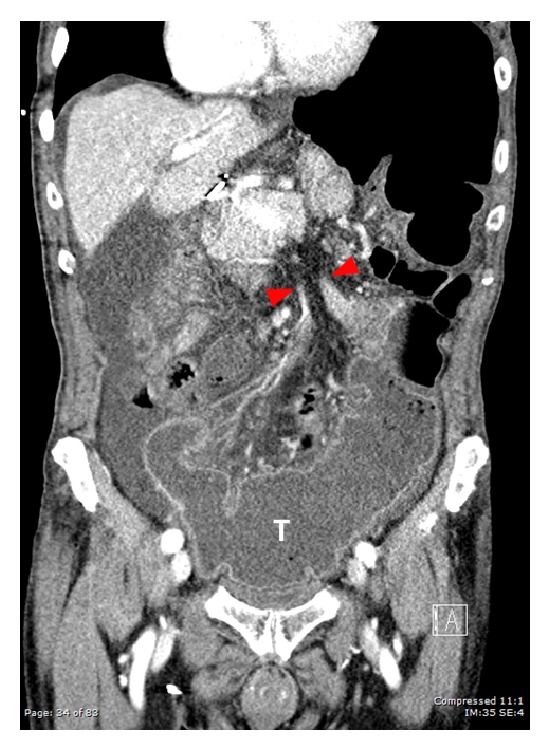
Coronal section from computer tomography imaging demonstrating the point of colonic volvulus (red arrows) within the transverse colon (T).

**Figure 2:**
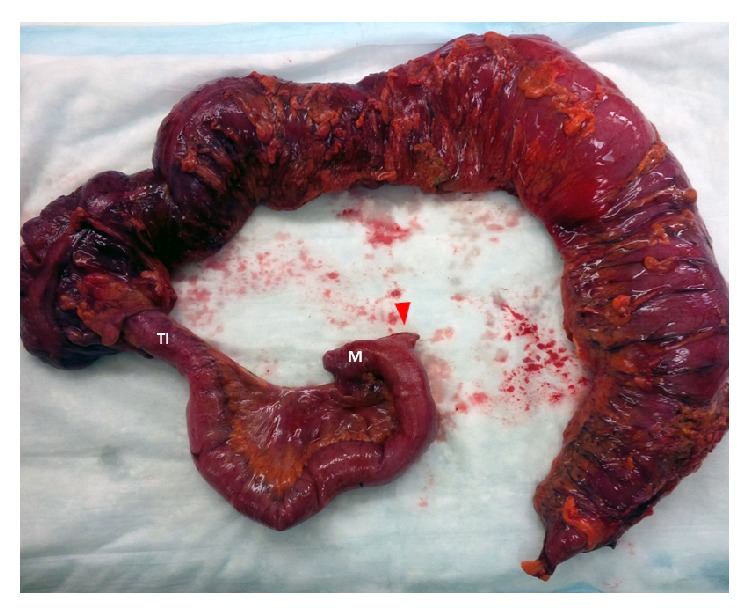
Resected specimen which included the ascending and transverse colon, terminal ileum (TI), and Meckel's diverticulum (M). The small bowel transection point was chosen just proximal to the Meckel's diverticulum (red arrow).

**Figure 3:**
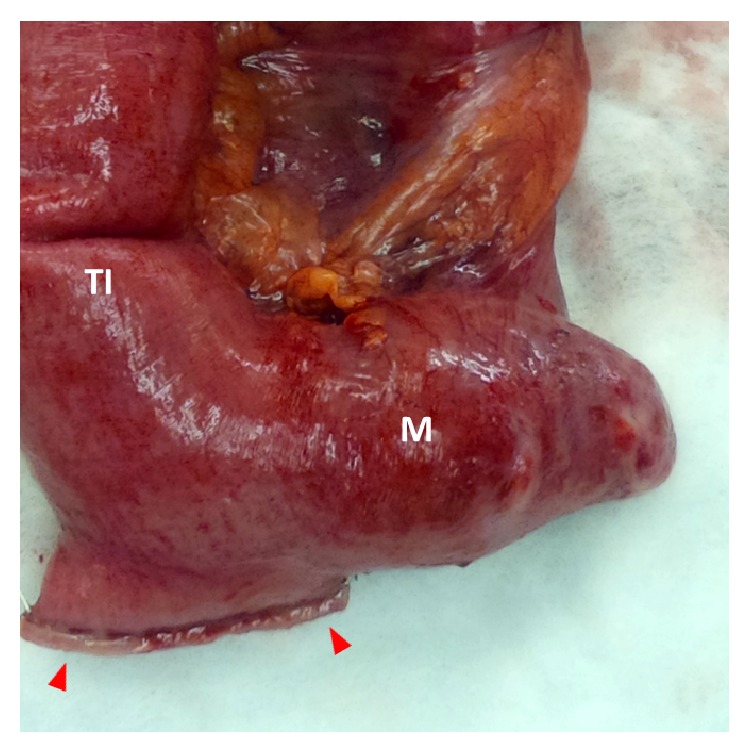
Gross specimen showing the Meckel's diverticulum (M) in relation to the terminal ileum (TI) and the proximal surgical staple line (red arrows). As shown, the Meckel's diverticulum had a relatively broad base; its measurements were 3.5 × 3.0 × 2.5 cm.

**Figure 4:**
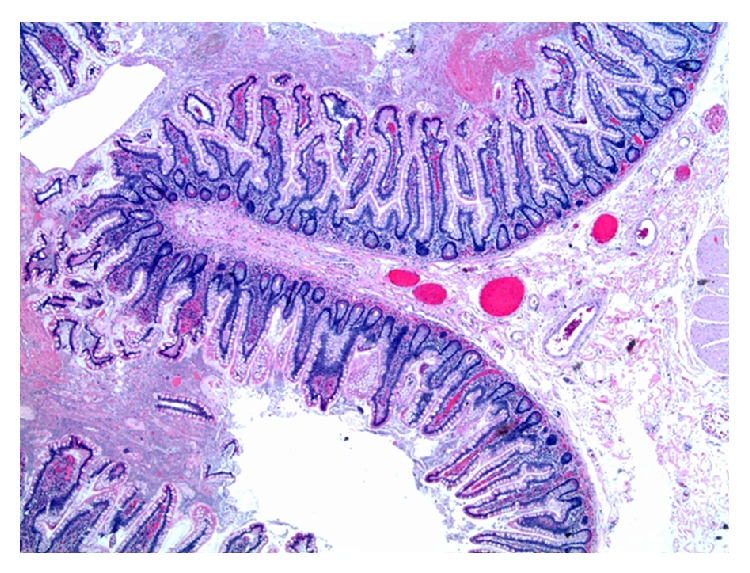
Low power view of the Meckel's diverticulum. Histological review did not reveal any aberrant tissue types.

**Figure 5:**
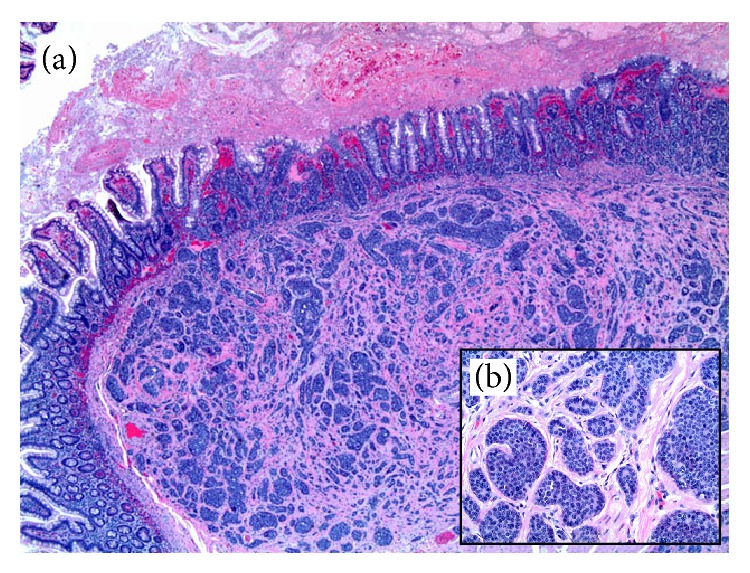
(a) Low power view of the incidental carcinoid found within the Meckel's diverticulum. The tumor was found to be 0.6 cm in its greatest dimension and showed invasion of the subserosa without involvement of the visceral peritoneum. Proximal and distal margins were uninvolved. Pathological staining was consistent with a pT2N0Mx carcinoid tumor. (b) High power view of the carcinoid tumor showing its acinar nests of uniform-appearing cells.

**Figure 6:**
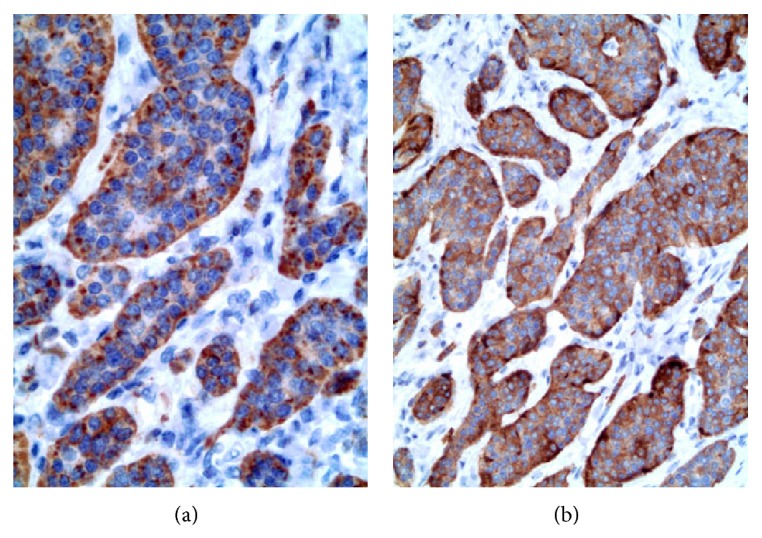
Histological analysis of this carcinoid found it to have a low proliferation index and therefore consistent with that of a low grade neuroendocrine tumor. Additional confirmatory immunohistological analysis found its cells to be positive for (a) synaptophysin and (b) chromogranin.

An 86-year-old male presented to our emergency department with acute onset abdominal pain. The patient's past medical and surgical history were noncontributory to his presenting condition. His vital signs were within normal limits and physical exam revealed tenderness within the upper midabdomen with accompanying guarding. Abdominal computer tomography revealed what appeared to be a volvulus involving the transverse colon (Figure 1) and the patient was taken urgently for surgery. Findings in the operating room indeed confirmed a volvulus involving the transverse colon, and the etiology appeared to be related to embryonic nonrotation. The small bowel was found to have right-sided predominance and the colon was found to be lacking in its usual retroperitoneal attachments. Following detorsion, the remainder of the patient's small bowel was examined, and in doing so, a Meckel's diverticulum was discovered in the terminal ileum (Figure 2). In inspecting the Meckel's diverticulum, it was readily apparent that its finding was unrelated to the colonic volvulus and that it was simply an incidental operative finding. The base of the diverticulum, however, was noted to be relatively wide (Figure 3) and the decision was made to include it as part of the resected specimen. A primary anastomosis was fashioned between the ileum and descending colon. Following surgery, the patient did well and was discharged home on the fifth post-operative day. Review of the final pathology confirmed the presence of a Meckel's diverticulum (Figure 4). Surprisingly, an incidental carcinoid tumor was found within the diverticulum (Figures 5(a) and 5(b)) and immunohistological analysis (Figures 6(a) and 6(b)) was confirmatory. Final pathology was consistent with that of a low grade neuroendocrine tumor with negative margins and 0 of 17 lymph nodes negative for nodal metastasis. Findings were discussed with the patient and no further clinical intervention was performed.

## 3. Discussion

Traditionally, pediatric surgical dogma cautiously advises that the finding of a congenital anomaly should raise the surgeon's awareness of other possible coexisting anatomic variations. Indeed, by experience, this proves to be true in the adult population as exemplified by our patient who had both a congenital colonic malrotation and a Meckel's diverticulum. In brief, intestinal malrotation is largely spoken of in pediatric surgery where children present early in life with bilious emesis. From the pediatric literature, it is believed that the majority of malrotations are uncovered during this period; however, review of the literature suggests that incidental or quiescent malrotation in the adult population is an increasingly common finding on routine imaging [3–6]. As such, there is far less controversy regarding the finding of quiescent malrotation as compared to the incidental Meckel's diverticulum, and surgical treatment of intestinal malrotation for adults is indicated when symptoms, such as bowel obstruction or volvulus, arise.

Meckel's diverticulum (MD) is a true diverticulum that results from persistence of the embryonic (omphalomesenteric) vitelline duct. Classically, a MD is found within 50 cm of the ileocecal valve and may contain heterotropic tissue, most commonly either gastric or pancreatic, in approximately half of specimens. In the adult population, it is well accepted that a symptomatic MD should be resected; however, the operative management of an asymptomatic incidentally found Meckel's remains controversial. This controversy is largely resolved in the pediatric population where it is strongly recommended that both symptomatic and asymptomatic MD should be resected as it is recognized that most patients develop symptoms within the first two years of life and the incidence of MD-related complications is inversely proportional to age [7]. Review of current literature finds there to be several anecdotal operative findings, a narrow base, long length, and palpable mucosal heterotopia, which have been used by some as guidelines for resection [8, 9]. Additional factors guiding resection of the asymptomatic MD include male gender and age less than fifty years [8, 10]. Conflicting discussions present within the literature have attempted to examine the risk benefit ratio for prophylactic resection in adults. By example, authors Cullen at al. were able to show that the operative morbidity and mortality for elective MD resection (2% and 1%) was significantly lower as compared to non-elective resection (12% and 2%) [2]. Conversely, when consideration is given to the overall low number of patients affected by MD, the argument presented by Zani et al., showing that 758 resections would have to be performed to prevent one death, remains arguably valid [11]. In our own experience, consideration for electively resecting an incidental MD needs to include the above-mentioned factors as well as assessment of the original inciting pathology requiring surgical intervention. The conversion of a clean surgical case not involving intestinal instrumentation to that of a contaminated one and the accompanying increased postoperative infection risk are of specific concern. Additional consideration likewise should be given to the surgical technique required for successful MD resection. A long MD with a narrow neck is amendable to simple diverticulectomy similar to that performed for an appendix. Conversely, a shorter MD with a broad neck frequently requires more extensive surgical effort and although wedge resection is a described technique, this approach is not always feasible and the possibility of a segmental bowel resection should be considered.

Meckel's diverticulum carcinoma is considered a rare finding (1–3%) and has not historically been a contributing factor in the literary discussion of incidental MD resection [12–14]. Regardless, in comparison to other ileal tumors, Meckel's diverticulum has been described as a high risk oncologic region with carcinoid tumors being the most common [12, 14]. In reviewing malignancies originating from Meckel's carcinoid (33%) and stromal tumors (leiomyosarcomas) (18%) occupy the highest percentage followed by gastric adenocarcinoma (12%) and, with increasing rarity, pancreatic adenocarcinoma, intraductal papillary mucinous neoplasms, gastrointestinal stromal tumors, and lymphomas [12, 14]. Gastrointestinal carcinoid tumors historically are not clinically recognizable until symptoms of obstruction or systemic carcinoid syndrome become present [8, 10]. Carcinoid syndrome, described as flushing, diarrhea, bronchial constriction with wheezing, and valvular heart disease, generally does not present until a patient has metastatic liver disease. These findings have important implications in that carcinoid tumors remain largely indolent while local and became clinically apparent only with advanced disease [15, 16]. Moreover, although it is commonly felt that the incidence of MD-related complications is inversely proportional to age, the incidence of MD-related carcinoid appears to proportionally increase with age [12]. Because carcinoid tumors are rarely detectable until they become clinically advanced, it is accordingly reasonable to argue that resection of incidentally found MD secondary to risk of carcinoid and other carcinomas is not entirely unreasonable. On review of the literature, there have been several case reports demonstrating discovery of MD carcinoma, but very few studies have discussed how these findings may impact the decision to resect an incidental MD [3]. Of particular significance, Thirunavukarasu et al. demonstrated increasing incidence of MD carcinoma over the last several decades lending support to resection and likewise showed increasing risk with age with a peak incidence occurring around the seventieth decade of life [12]. Indeed, argument for resection is further supported with the finding of local disease in the majority of MD-related cases, thus making surgical intervention curative.

The resection of incidentally found Meckel's diverticulum in adults remains a controversial topic in the surgical literature. In our patient, removal of an incidental Meckel's diverticulum has led to the discovery of a low grade carcinoid tumor. Commonly cited operative findings used in favor of resection have traditionally included a narrow base, long diverticulum length, and palpable mucosal heterotopia. More recent literature has raised awareness of the small, but unmistakable, accompanying risk of malignancy associated with the Meckel's diverticulum. Arguably, although resection is not without risk, the clinical consequence associated with leaving an asymptomatic Meckel's may be greater. In combination with overall increased patient longevity and the risk of Meckel's diverticulum carcinoma, we accordingly suggest that the adult guidelines for the management of asymptomatic incidental Meckel's resection be reexamined.
